# Characterization of the human ridged and non-ridged skin: a comprehensive histological, histochemical and immunohistochemical analysis

**DOI:** 10.1007/s00418-018-1701-x

**Published:** 2018-08-11

**Authors:** A. Vela-Romera, V. Carriel, M. A. Martín-Piedra, J. Aneiros-Fernández, F. Campos, J. Chato-Astrain, N. Prados-Olleta, A. Campos, M. Alaminos, I. Garzón

**Affiliations:** 1 Tissue Engineering Group, Department of Histology, Faculty of Medicine, University of Granada, Avenida de la Investigación 11, Torre A, Planta 5, 18016 Granada, Spain; 2 PhD Program in Biomedicine, Escuela de Posgrado, University of Granada, Granada, Spain; 3 Instituto de Investigación Biosanitaria ibs.GRANADA, Granada, Spain; 4 Department of Pathology, San Cecilio University Hospital, Granada, Spain; 5 Orthopedic Surgery Department, Virgen de las Nieves University Hospital, Granada, Spain; 6 Department of Surgery, University of Granada, Granada, Spain

**Keywords:** Skin, Histology, Immunohistochemistry, Histochemistry, Quantitative analysis, Non-ridged skin, Ridged skin, Glabrous skin, Palmoplantar skin

## Abstract

The structure of the human skin is directly dependent on its location and the mechanical forces to which it is subjected. In the present work, we have performed a comprehensive analysis of the human ridged and non-ridged skin to identify the differences and similarities between both skin types. For this purpose, human skin samples were obtained from dorsal hand skin (DHS), palmar hand skin (PHS), dorsal foot skin (DFS) and plantar foot skin (PFS) from the same cadaveric donors. Histological, histochemical and semiquantitative and quantitative immunohistochemical analyses were carried out to evaluate the epidermis, dermis and basement membrane. Results show that the epithelial layer of ridged skin had larger cell number and size than non-ridged skin for most strata. Melanocytes and Langerhans cells were more abundant in non-ridged skin, whereas Merkel cells were preferentially found in ridged skin. The expression pattern of CK5/6 was slightly differed between non-ridged and ridged skin. Involucrin expression was slightly more intense in non-ridged skin than in ridged skin. Collagen was more abundant in foot skin dermis than in hand skin, and in ridged skin as compared to non-ridged skin. Elastic fibers were more abundant in DHS. Biglycan was more abundant in foot skin than in hand skin. No differences were found for blood and lymphatic vessels. The basement membrane laminin was preferentially found in foot skin. These results revealed important differences at the epithelial, dermal and basement membrane levels that could contribute to a better knowledge of the human skin histology.

**Electronic supplementary material:**

The online version of this article (10.1007/s00418-018-1701-x) contains supplementary material, which is available to authorized users.

## Introduction

The skin is a complex organ whose functions are tightly related to its histological structure. In fact, the skin structure may vary according to the anatomical area in which it is found (Mills 2007; Khavkin and Ellis 2011). In this context, two types of skin can be found according to its thickness and characteristics: the pressure-resistant ridged skin found in feet soles and hand palms (also called thick, hairless or glabrous skin), and the non-ridged skin found at most of the body surface (also called thin or hairy skin) (Khavkin and Ellis 2011; Kanitakis 2002; Geneser 1986). Although both types of skin may be affected by common skin conditions, certain diseases tend to preferentially affect only one type of skin. Specifically, palmoplantar skin is affected by a number of conditions such as palmoplantar keratoderma that do not commonly affect other anatomical areas (Van Steensel 2010; Sakiyama and Kubo 2016). In addition, the loss of palmoplantar skin areas due to trauma, tumors or burns is an important medical challenge (Banis 2001; Uroskie and Colen 2001; Donato et al. 2000) that is often treated by transference of free skin grafts from other areas to the palmoplantar area (Engelhardt et al. 2011).

The histological structure of the human skin has been extensively studied (Uemura et al. 2016). However, very few studies are focused on determining the main characteristics of ridged skin as compared to non-ridged skin, and the differences and similarities between non-ridged and ridged skin are not well known. Therefore, a deeper knowledge of ridged skin is necessary for a better understanding of pathological conditions affecting the human ridged skin to achieve an appropriate treatment of diseases affecting this area. In addition, it is important to identify the differences between the ridged skin found at the hand palms and at the feet soles.

In the present work, we have performed a comprehensive histological, histochemical and immunohistochemical analysis of the human ridged skin as compared to non-ridged skin of hands and feet from the same individuals to shed light on the main characteristics of ridged skin and to determine the differences between both skin types.

## Materials and methods

### Human skin samples

Human samples of dorsal hand skin (DHS), palmar hand skin (PHS), dorsal foot skin (DFS) and plantar foot skin (PFS) were obtained from six cadaveric donors (< 24 h) whose age ranged between 25 and 70 years (average 48.8 ± 21.0) and did not have any previously known skin disease. All samples’ ridged and non-ridged skin was procured from the same donors for consistency and to avoid interpersonal variations. In each case, full-thickness skin biopsies with an average size of 1 cm^2^ were obtained using a surgical knife from: (1) the hand skin allocated dorsally to metacarpals (DHS) and palmarly to metacarpals, at the level of the metacarpal heads (PHS) and (2) the foot skin allocated dorsally to metatarsals (DFS) and plantarly to metatarsals, at the level of the metatarsal heads (FPS). Each type of skin was analyzed in all donors and averages and standard deviations were calculated for each type of analysis to compensate individual differences. An authorization was obtained from the Department of Anatomy of the Medical School of the University of Granada, which approved the study.

### Histological analysis

Once obtained, each sample was divided in three fragments, which were fixed, respectively, in 4.0% w/v neutral buffered formaldehyde (for light microscopy, histochemistry and immunohistochemistry for Melan-A, CD1A, Smoothelin, SMA-ACT, CD-31 and D2-40), methacarn fixative consisting of 60% methanol, 30% chloroform and 10% glacial acetic acid (for immunohistochemistry for CK5/6, CK7, CK10, CK20, claudin-1, DSPK 1-2, involucrin, filaggrin, laminin, collagen types I, III and IV, decorin, biglycan and versican) and 2.5% glutaraldehyde (for electron microscopy). Samples fixed in formaldehyde or methacarn were embedded in paraffin, and 5 µm tissue sections were obtained for hematoxylin and eosin (H&E) staining using routine methods. H&E stained sections were photographed using a Nikon Eclipse 90i light microscope (Nikon Corp., Tokyo, Japan) and thickness of each epithelial stratum was quantified in each sample using a scale as a reference. For this purpose, 30 equidistant vertical lines were drawn in each histological image using a predesigned template, and height of each cell layer was assessed at each point. This way, both the rete ridges and the papillae were measured and the average of all 30 measured was calculated.

### Immunohistochemistry

Specific proteins of the skin epidermis, dermis and basement membrane were detected by immunohistochemistry using primary antibodies anti-Melan-A, CD1A, cytokeratins CK5/6, CK7, CK10 and CK20, claudin-1, desmoplakin 1/2, involucrin, filaggrin, collagens type I, III and IV, decorin, biglycan, versican, smoothelin, SMA-ACT, CD-31, D2-40 and laminin. Tissue sections were dewaxed and rehydrated and antigen retrieval was carried out at 98 °C only for formalin-fixed tissues. Endogenous peroxidase was blocked with 3% of H_2_O_2_ in PBS and unspecific Ab binding was prevented with horse serum (Vector Laboratories). Primary antibodies were added and samples were incubated in a humid chamber (see Supplementary Table S1 for technical details). Secondary antibodies conjugated with peroxidase were used and the Ag–Ab reaction was visualized using DAB. Immunostaining was contrasted with Harris hematoxylin. Images were obtained using a Nikon Eclipse 90i light microscope (Nikon Corp., Tokyo, Japan) and the immunohistochemical signal intensity was semiquantitatively assessed as strong (+++), mild (++), slight (+) or negative (−) as previously described (Garcia-Martinez et al. 2017). Samples immunostained with anti-Melan A antibodies were used to quantify the number of melanocyte cells per 100 µm of length of the epidermis in each sample, CD1A was used to quantify Langerhans cells and CK20 was used as a marker of Merkel cells.

### Histochemistry

Identification of the key components of the extracellular matrix of the human dermis—collagen, elastic and reticular fibers and proteoglycans—was carried out by histochemistry. For this purpose, dewaxed and rehydrated tissue sections were stained using the Fontana-Masson Picrosirius (FMP) method for melanin, collagen and morphology as previously described (Carriel et al. 2011). The main components of the dermis extracellular matrix (ECM) were histochemically identified by orcein staining for elastic fibers (Oliveira et al. 2013), Gomori’s reticulin metal reduction technique for reticular fibers and alcian blue staining for proteoglycans, following previously established protocols (Kiernan 1999). The FMP method was also used to determine the area occupied by the epithelial cells in each epidermal stratum.

Quantification of the histochemical signal as determined by percentage of area occupied by positively stained structures and intensity of the signal was performed in each sample for each histochemical method using the ImageJ software (National Institutes of Health, USA) as previously described (Carriel et al. 2013, 2017). Briefly, intensity was determined by randomly selecting ten points of each image using the multi-point tool of the software, and the average intensity was calculated by subtracting the white signal to the value calculated by the program. Area was determined by converting the images to binary (black and white) and using the threshold function to isolate the colors of interest. Then, the ‘area fraction’ function was used to automatically calculate the percentage of black elements (positive areas) present in a specific area of the tissue section. All images were taken and analyzed using exactly the same conditions (exposition time, white balance, background, etc.) with the Nikon NIS-Elements software. To analyze the cell area of keratinocytes found at each stratum and the number of keratinocytes per 100 µm of epithelial length, the Nikon NIS-Elements software was used. In this case, samples stained with the FMP method were used to obtain a better definition of the cellular contour.

### Electron microscopy analysis

For scanning electron microscopy (SEM), samples fixed in 2.5% glutaraldehyde were dehydrated, prepared for critical point, dried and mounted on aluminum stubs, sputter-coated with gold and examined in a Quanta 200 scanning electron microscopy (FEI, Eindhoven, The Netherlands).

For transmission electron microscopy (TEM), samples fixed in 2.5% glutaraldehyde were postfixed in 1% osmium tetroxide, dehydrated in increasing concentrations of acetone (30%, 50%, 70%, 95% and 100%), embedded in Spurr’s resin and cut in ultrathin sections, using an ultramicrotrome. Ultrathin sections were stained with aqueous uranyl acetate and lead citrate and examined with aEM902 transmission electron microscope (Carl Zeiss Meditec, Inc., Oberkochen, Germany).

### Statistical analysis

First, averages and standard deviations were obtained for each type of skin: DHS, PHS, DFS and PFS, for hand skin (DHS and PHS), foot skin (DFS and PFS), ridged skin (PHS and PFS) and non-ridged skin (DHS and DFS) for the following quantitative parameters: thickness of each stratum, cell area, number of cells found at each stratum, intensity and area of structures stained with each histochemical method. Second, results obtained for each type of skin were compared between two different groups using the Mann–Whitney *U* test. *p* values below 0.05 were considered as statistically significant for the double-tailed tests.

## Results

### Characterization of the human skin epidermis

#### Histological characterization by light microscopy

**Fig. 1:**
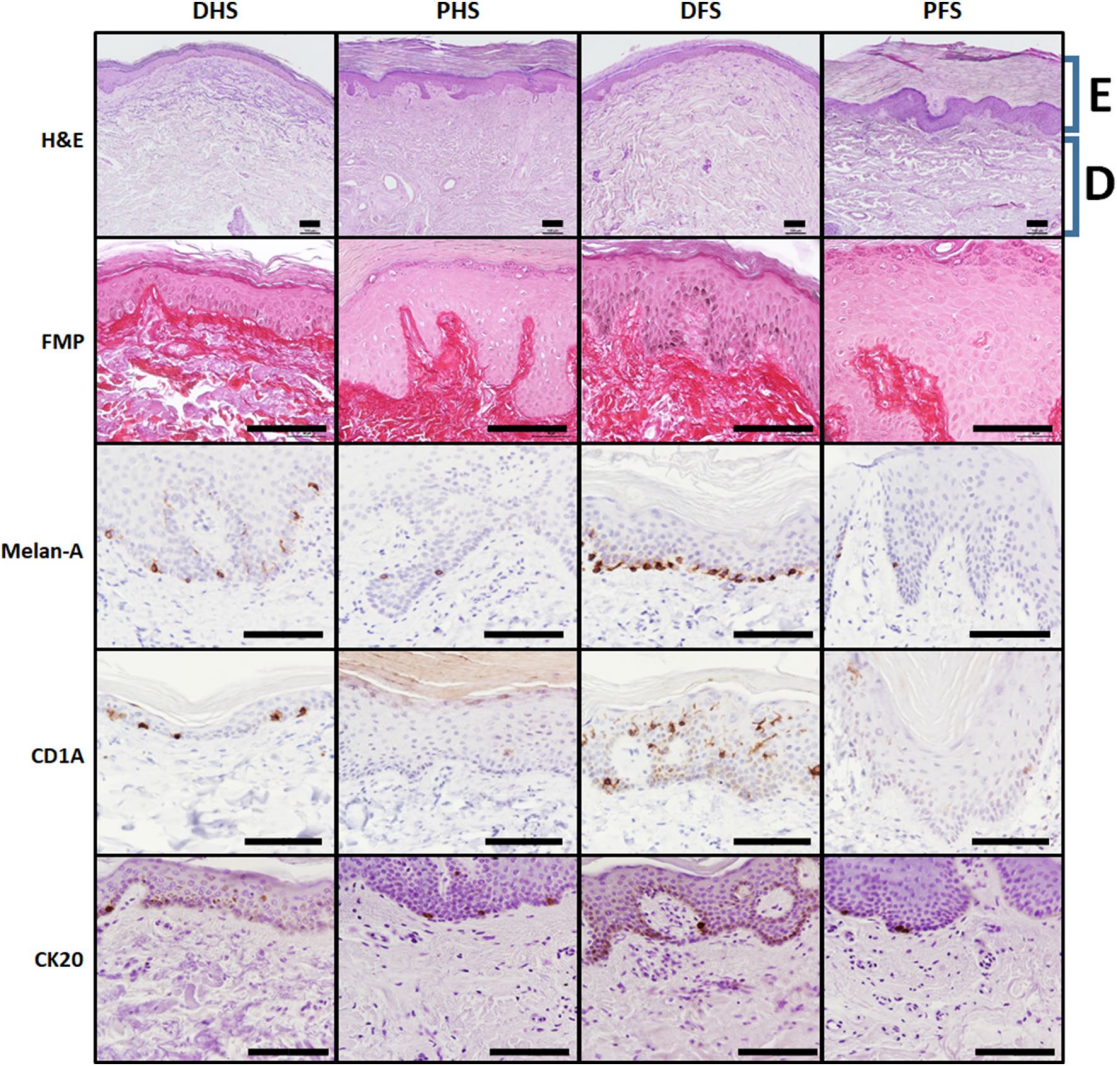
Histological, histochemical and immunohistochemical analysis of epidermal cells in the four skin types analyzed in this work. H&E: hematoxylin–eosin histological analysis showing a global vision of each skin type. E: epidermal layer, D: dermal layer; FMP: Fontana-Masson-Picrosirius histochemical method showing the epidermal cells in pink color and the dermal layer in red. Melanin appears in black color at the basal layers of some of the samples due to the argentaffin reaction of the FMP method; Melan-A, CD1A and CK20: immunohistochemical analyses for melanocytes, Langerhans and Merkel cells, respectively, showing the presence of these cell types in brown color. *DHS* dorsal hand skin, *PHS* palmar hand skin, *DFS* dorsal foot skin, *PFS* plantar foot skin. Scale bars: 100 µm

Histological analysis of skin samples stained with hematoxylin and eosin showed that all skin types consisted of an epidermal layer and a subjacent dermal layer (Fig. 1), but substantial differences existed among the four skin types. At the epithelial layer, non-ridged dorsal skin of the human hand and foot is characterized by a stratified keratinized epithelium with 8–12 epithelial cell layers below a thin corneous stratum (stratum corneum), whilst ridged palmar and plantar skin showed a high number of epithelial layers that ranged between 30 and 60 layers approximately, and a thick corneous stratum. Quantification of the thickness of the epithelial layers showed significant differences among the groups compared (Table 1). In general, palmar and plantar skin was significantly thicker than DFS and DHS, and dorsal skin corresponding to hand and foot was thinner than palm and plantar skin, respectively. In addition, plantar skin was significantly thicker than palmar skin (*p* < 0.05), but DFS was similar to DHS.

**Table 1:** Quantitative analysis of thickness, cell number and cell area of human skin epidermis cells

	Thickness of the strata					Cell area				Number of cells per 100 µm lineal						
	Epidermis	Basal layer	Spinous layer	Granular layer	Horny layer	Basal layer	Spinous layer	Granular layer	Horny layer	Basal layer	Spinous layer	Granular layer	Horny layer	Melanocytes	Langerhans cells	Merkel cells
Dorsal foot skin (DFS)	116.5 ± 85.2	13.4 ± 3.9	45.9 ± 37.3	7.9 ± 8.5	35.9 ± 27.8	59.8 ± 11.1	91.0 ± 41.6	83.4 ± 52.7	31.8 ± 5.1	22.3 ± 3.6	44.3 ± 17.9	8.4 ± 4.1	101.4 ± 11.5	2.6 ± 2.1	2.6 ± 1.6	0.03 ± 0.03
Dorsal hand skin (DHS)	75.1 ± 6.5	11.6 ± 0.0	22.1 ± 6.5	4.2 ± 0.1	33.1 ± 1.6	57.6 ± 5.5	74.2 ± 30.3	61.5 ± 19.2	32.5 ± 11.4	20.3 ± 2.0	31.0 ± 3.9	7.3 ± 2.4	114.3 ± 26.1	1.9 ± 0.2	1.2 ± 0.2	0.07 ± 0.04
Plantar foot skin (PFS)	724.4 ± 232.0	15.8 ± 4.7	126.7 ± 60.1	29.7 ± 14.4	487.7 ± 160.2	70.7 ± 16.9	195.5 ± 45.1	217.4 ± 49.9	128.7 ± 31.2	22.3 ± 3.5	63.1 ± 20.8	13.4 ± 6.0	392.7 ± 136.7	0.7 ± 0.5	1.1 ± 1.2	0.37 ± 0.26
Palmar hand skin (PHS)	264.0 ± 30.6	13.0 ± 2.5	46.6 ± 4.5	13.1 ± 0.8	148.5 ± 36.4	58.3 ± 3.3	129.7 ± 29.8	135.5 ± 53.6	66.5 ± 19.6	22.2 ± 3.0	36.8 ± 5.0	11.1 ± 5.0	252.1 ± 129.2	0.4 ± 0.1	0.1 ± 0.0	0.36 ± 0.10
Foot	448.1 ± 361.4	90.0 ± 64.4	19.8 ± 16.2	91.5 ± 53.1	282.3 ± 262.3	65.7 ± 15.0	148.0 ± 68.5	156.5 ± 85.2	84.7 ± 55.3	22.3 ± 3.3	54.5 ± 21.0	11.1 ± 5.6	260 ± 180.4	1.7 ± 1.8	1.8 ± 1.5	0.20 ± 0.25
Hand	169.5 ± 103.0	34.4 ± 14.1	8.6 ± 4.8	57.2 ± 42.9	90.8 ± 66.2	58.0 ± 4.2	102.0 ± 40.7	98.5 ± 54.4	49.5 ± 23.4	21.2 ± 2.6	33.9 ± 5.2	9.2 ± 4.2	183.2 ± 113.4	1.1 ± 0.8	0.7 ± 0.6	0.21 ± 0.17
Ridged skin	540.2 ± 294.6	94.7 ± 61.0	23.1 ± 13.7	115.9 ± 35.4	352.0 ± 213.0	65.7 ± 14.3	169.2 ± 50.8	184.7 ± 64.3	103.8 ± 41.3	22.3 ± 3.1	52.6 ± 20.8	12.5 ± 5.5	336.5 ± 145.7	0.6 ± 0.4	0.7 ± 1.0	0.37 ± 0.19
Non-ridged skin	98.1 ± 64.2	35.3 ± 29.5	6.3 ± 6.3	33.9 ± 20.4	34.7 ± 19.8	58.8 ± 8.6	83.5 ± 35.9	73.7 ± 40.7	32.1 ± 7.9	21.4 ± 3.0	38.4 ± 14.7	7.9 ± 3.3	107.1 ± 19.2	2.3 ± 1.6	2.0 ± 1.3	0.05 ± 0.04
DFS vs. DHS	0.6213	0.6213	0.3231	0.6213	0.6213	0.6213	0.6213	0.3231	0.6242	0.3231	0.1383	1.000	0.8064	0.6213	0.0481*	0.12133
DFS vs. PFS	0.0062*	0.4652	0.0285*	0.0176*	0.0062*	0.2733	0.0106*	0.0062*	0.0061*	0.8551	0.1441	0.1441	0.0061*	0.0465*	0.0758	0.0084*
PFS vs. PHS	0.0101*	0.3909	0.0101*	0.0862	0.0101*	0.3909	0.0319*	0.0862	0.0101*	1.000	0.0101*	1.000	0.1981	0.6198	0.2109	0.6198
DHS vs. PHS	0.018*	1.0000	0.018*	0.018*	0.018*	1.000	0.0180*	0.0180*	0.0795	0.2367	0.2367	0.2367	0.0795	0.0180*	0.0180*	0.0179*
Foot vs. hand	0.1365	0.3209	0.0471*	0.3209	0.3209	0.4421	0.2060	0.2060	0.1860	0.4421	0.0018*	0.7780	0.5629	0.7216	0.0611	0.5895
Ridged vs. non-ridged	0.0004*	0.4134	0.0032*	0.0014*	0.0002*	0.4470	0.0002*	0.0001*	0.0004*	0.4002	0.1564	0.0535	0.0004*	0.0009*	0.0115*	0.0005*

Thickness of each epithelial stratum is shown in micrometers (average ± standard deviation), whereas the cell area is shown in square micrometers (average ± standard deviation). The last rows correspond to statistical *p* values for the comparison of two specific skin types

*DHS* dorsal hand skin, *PHS* palmar hand skin, *DFS* dorsal foot skin, *PFS* plantar foot skin

* * p* values below 0.05 are labeled with asterisks

When specific epithelial layers were compared using FMP-stained samples (Fig. 1), we found no differences for the basal stratum among the different skin types. However, thickness of the spinosum, granulosum and corneum strata was higher in ridged skin than in non-ridged skin, especially in the plantar region.

These results are in agreement with data obtained for the area of cells corresponding to each stratum as determined by the FMP method. As shown in Table 1, the area of cells at the basal stratum was similar in all skin samples. However, spinosum and granulosum strata keratinocytes and corneum stratum corneocytes of ridged skin were more than twice as large as the same cells found in non-ridged skin, with differences being statistically significant (*p* < 0.05). On the other hand, quantification of the number of cells found per unit of length of skin revealed that the number of cells tended to be similar among the different skin types compared for basal and granulosum strata, and statistical differences were found for the spinosum stratum only for the comparison of foot skin vs. hand skin, especially when ridged skin of these two regions was considered. The number of corneocytes at the horny layer was significantly higher in ridged skin than in non-ridged skin, especially in PFS.

When other epithelial cell types were quantified at the epidermis of the different samples, we found significant differences among several types of skin (Fig. 1; Table 1). In short, the number of melanocytes found per unit of length (as determined by Melan-A) was about four times higher in non-ridged skin than in ridged skin, for both the foot and the hand, and were especially abundant in DFS. This is in agreement with our FMP results showing positive argentaffin reaction at the basal layers of DHS and, especially, DFS, suggesting that melanin was more abundant in these samples. Regarding Langerhans cells as determined by CD1A, non-ridged skin had almost three times the number of cells found in ridged skin, and DFS had significantly more cells than DHS (*p* < 0.05). Finally, Merkel cells as determined by CK20 immunohistochemistry were significantly more abundant in ridged skin as compared to non-ridged skin.

#### Histological characterization by electron microscopy

**Fig. 2:**
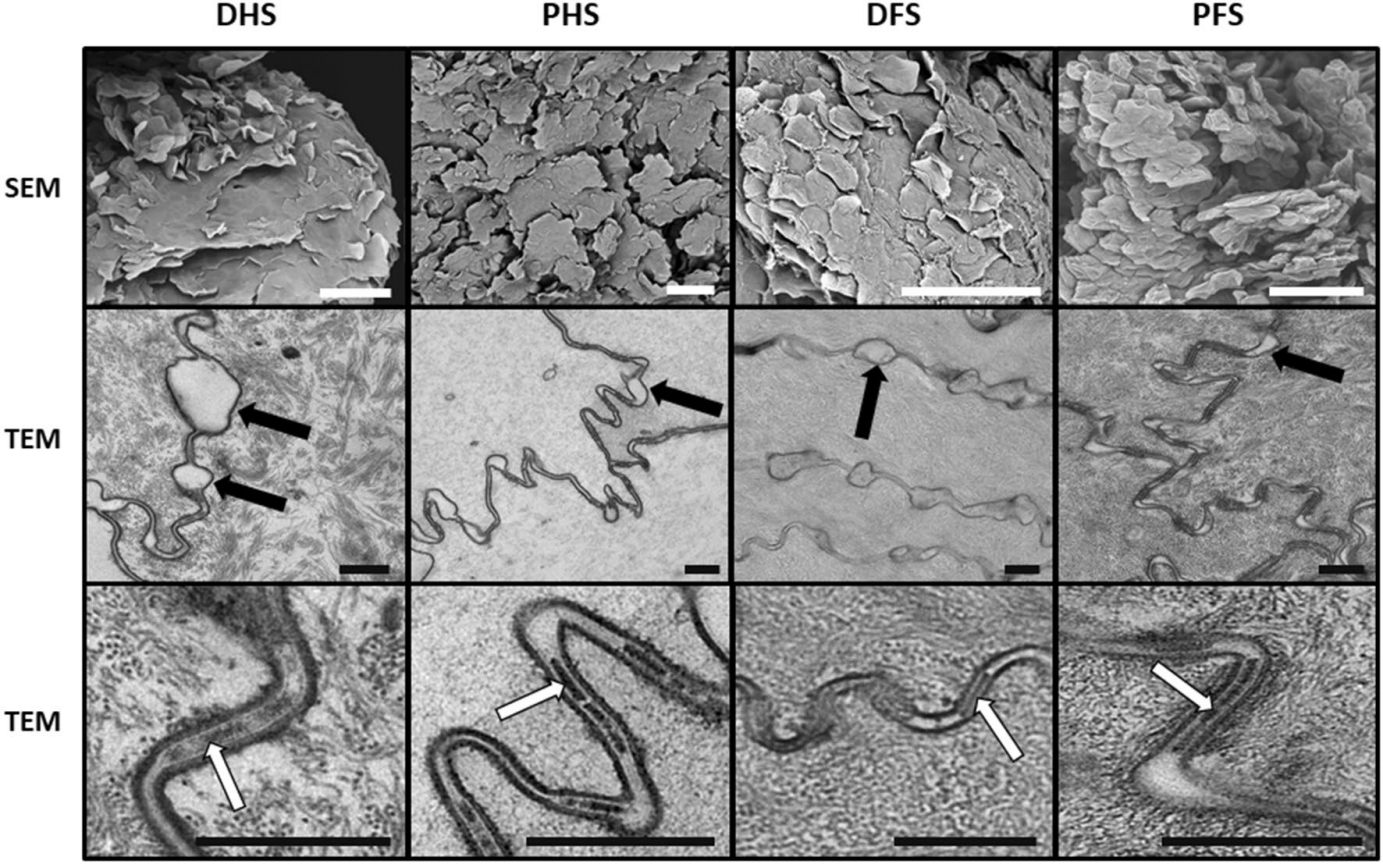
Electron microscopy evaluation of the four skin types analyzed in this work. SEM: scanning electron microscopy showing superficial desquamation of epithelial corneocytes in the four types of skin. TEM: transmission electron microscopy analysis of skin corneocytes. These cells showed numerous intercellular empty spaces corresponding to areas where cells begin to separate from each other at the onset of the desquamation process (black arrows). White arrows correspond to corneodesmosomes found at the intercellular space between neighboring cells. *DHS* dorsal hand skin, *PHS* palmar hand skin, *DFS* dorsal foot skin, *PFS* plantar foot skin. Scale bars: 100 µm for SEM and 500 nm for TEM

To further characterize the most apical epithelial layers of each skin type, we used electron microscopy (Fig. 2). First, SEM showed that the surface of all skin types consisted of numerous irregular or polygonal cells that tended to detach from adjacent cells, but very few differences were found among the skin types. The process of superficial desquamation of the cells did not show any differences among samples. Second, TEM analysis of the stratum corneum showed that corneocytes were very similar in the four types of skin compared, with all cells showing very few intracellular organelles and abundant intracellular keratin fibers. Cells were devoid of desmosomes and tight junctions, but showed numerous interdigitations between neighboring cells. Empty spaces were found scattered all along the cell–cell contact area, suggesting that cells may begin to separate from each other at those specific points at the beginning of the physiological desquamation process of superficial cells. Moreover, corneodesmosomes were very abundant at the intercellular space found between neighboring cells, but no differences were detected among samples.

#### Immunohistochemical characterization of the skin epidermis

**Fig. 3:**
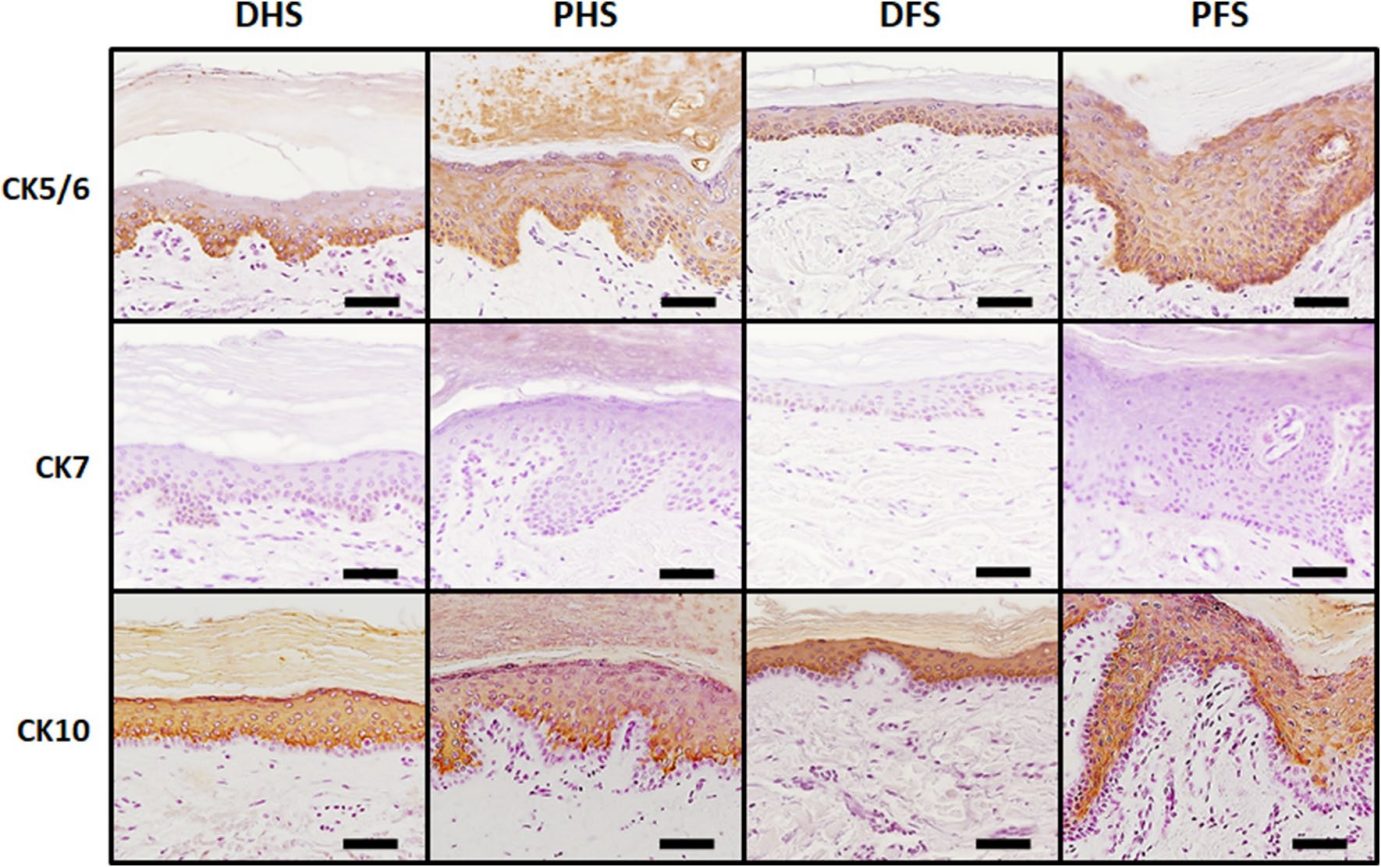
Immunohistochemical analysis of cytokeratins CK5/6, CK7 and CK10 in the epidermis of the four skin types analyzed in this work. The image shows strong CK5/6 immunoreaction (identified in brown) at the basal layer of the epidermis of all samples, whereas suprabasal signal was more evident for PHS and PFS as compared to DHS and DFS. CK7 was negative for all samples, whereas CK10 showed strong positive reaction at suprabasal layers of all samples, with no differences among samples. *DHS* dorsal hand skin, *PHS* palmar hand skin, *DFS* dorsal foot skin, *PFS* plantar foot skin. Scale bars: 50 µm

To identify specific proteins at the epithelial layer of ridged and non-ridged skin, we carried out immunohistochemical analyses. First, detection of cytokeratin CK5/6 revealed strong positive expression (+++) at the basal epithelial layer of the four types of skin, with no differences among samples at the basal stratum, but ridged skin tended to show higher expression (++) than non-ridged skin (+) at suprabasal layers (Fig. 3; Table 2). In contrast, CK7 was negative (−) in all epithelial layers of the four skin types. Finally, CK10 was strongly positive (+++) in suprabasal layers of all skin samples and negative (−) in basal layers, and no substantial differences were found among the different skin samples analyzed in this work.

**Table 2:** Immunohistochemical analysis of relevant components of the human skin epidermis, dermis and basement membrane

Marker	DHS	PHS	DFS	PFS
Cytokeratin 5/6	(BS) +++/(SB) +	(BS) +++/(SB) ++	(BS) +++/(SB) +	(BS) +++/(SB) ++
Cytokeratin 7	(BS) −/(SB) −	(BS) −/(SB) −	(BS) −/(SB) −	(BS) −/(SB) −
Cytokeratin 10	(BS) −/(SB) +++	(BS) −/(SB) +++	(BS) −/(SB) +++	(BS) −/(SB) +++
Claudin-1	(BS) −/(SB) +++	(BS) −/(SB) +++	(BS) −/(SB) +++	(BS) −/(SB) +++
Desmoplakin 1/2	(BS) −/(SB) +++	(BS) −/(SB) +++	(BS) −/(SB) +++	(BS) −/(SB) +++
Involucrin	(BS) −/(SB) +++	(BS) −/(SB) ++	(BS) −/(SB) +++	(BS) −/(SB) ++
Filaggrin	(BS) −/(SB) ++	(BS) −/(SB) ++	(BS) −/(SB) ++	(BS) −/(SB) ++
Collagen I	++	++	++	+++
Decorin	++	++	++	++
Biglycan	+	+	++	+++
Versican	++	+	++	++
Laminin	+	+	++	++
Collagen III	+++	+++	+++	+++
Collagen IV	+++	+++	+++	+++

(BS): Expression at the basal layer of the epidermis. (SB): Expression at the suprabasal layers of the epidermis. The signal intensity was semiquantitatively assessed as strong (+++), mild (++), slight (+) or negative (−)

**Fig. 4:**
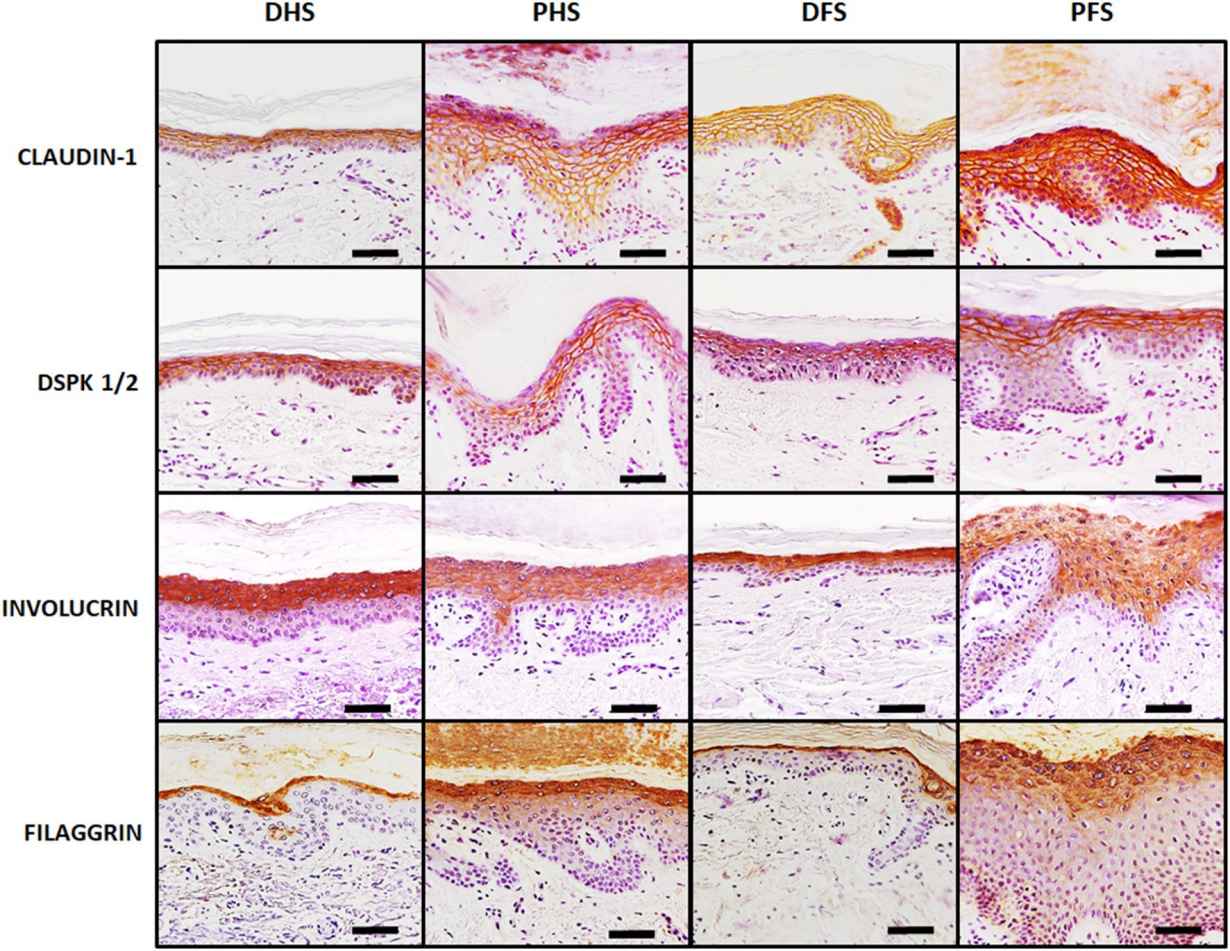
Immunohistochemical analysis of relevant intercellular junction proteins (claudin-1 and desmoplakin 1/2) and terminal differentiation markers (filaggrin and involucrin) in the epidermis of the four skin types analyzed in this work. Both intercellular junction proteins were strongly positive (in brown) only at suprabasal epithelial layers, with no differences among samples. Involucrin was strongly positive at suprabasal layers of non-ridged skin and mildly positive at suprabasal layers of ridged skin, whereas filaggrin was mildly positive at suprabasal layers of all samples. *DHS* dorsal hand skin, *PHS* palmar hand skin, *DFS* dorsal foot skin, *PFS* plantar foot skin. Scale bars: 50 µm

Analysis of intercellular junctions as determined by immunohistochemistry revealed strong positive expression (+++) of both claudin-1 and desmoplakin 1/2 in suprabasal layers of all types of skin, especially at the stratum spinosum, with very few differences among samples. As shown in Fig. 4 and Table 2, ridged and non-ridged skin had similar expression of these cell–cell junction markers.

Detection of the epithelial differentiation markers involucrin and filaggrin demonstrated that both markers were positive in all samples, although the expression patterns were different among skin types (Fig. 4; Table 2). First, involucrin was positive in suprabasal layers, especially at the stratum granulosum and at the most apical layers of the stratum spinosum, with no signal at the corneous stratum. Expression of this protein was more intense in non-ridged skin (+++) than in ridged skin (++). For filaggrin, expression was suprabasal, but restricted to the stratum granulosum (++), with slight expression at the stratum corneum. For involucrin and filaggrin, the signal found in non-ridged skin tended to be sharp and well delimited, whereas ridged skin showed a more diffuse pattern distributed throughout a higher number of epithelial cell layers.

### Characterization of the human skin dermis

#### Histochemical quantification of skin dermis components

**Fig. 5:**
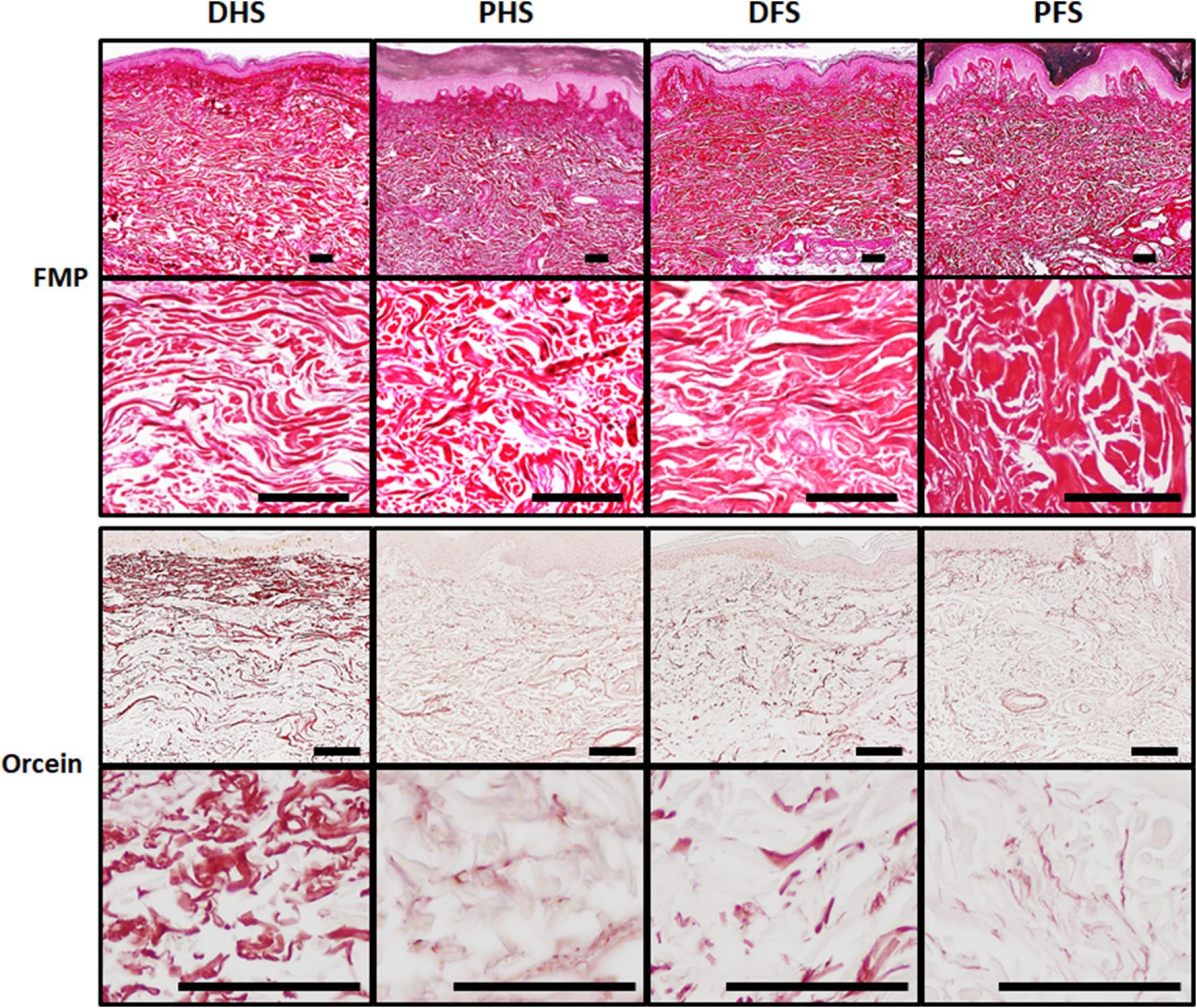
Histochemical analysis of collagen and elastic fibers in the dermis of the four skin types analyzed in this work as determined by the Fontana-Mason-Picrosirius (FMP) and orcein methods, respectively. For the FMP method, all samples showed intense positive histochemical reaction (in red) revealing that the content of collagen fibers was very high in all tissue types, although differences existed among samples. For the orcein method, DHS showed significantly higher amount of elastic fibers (in brownish red) than the rest of the samples. *DHS* dorsal hand skin, *PHS* palmar hand skin, *DFS* dorsal foot skin, *PFS* plantar foot skin. Top panels correspond to low-magnification images of each skin type, whereas lower panels show higher magnifications of the same sample type. Scale bars: 100 µm

**Fig. 6:**
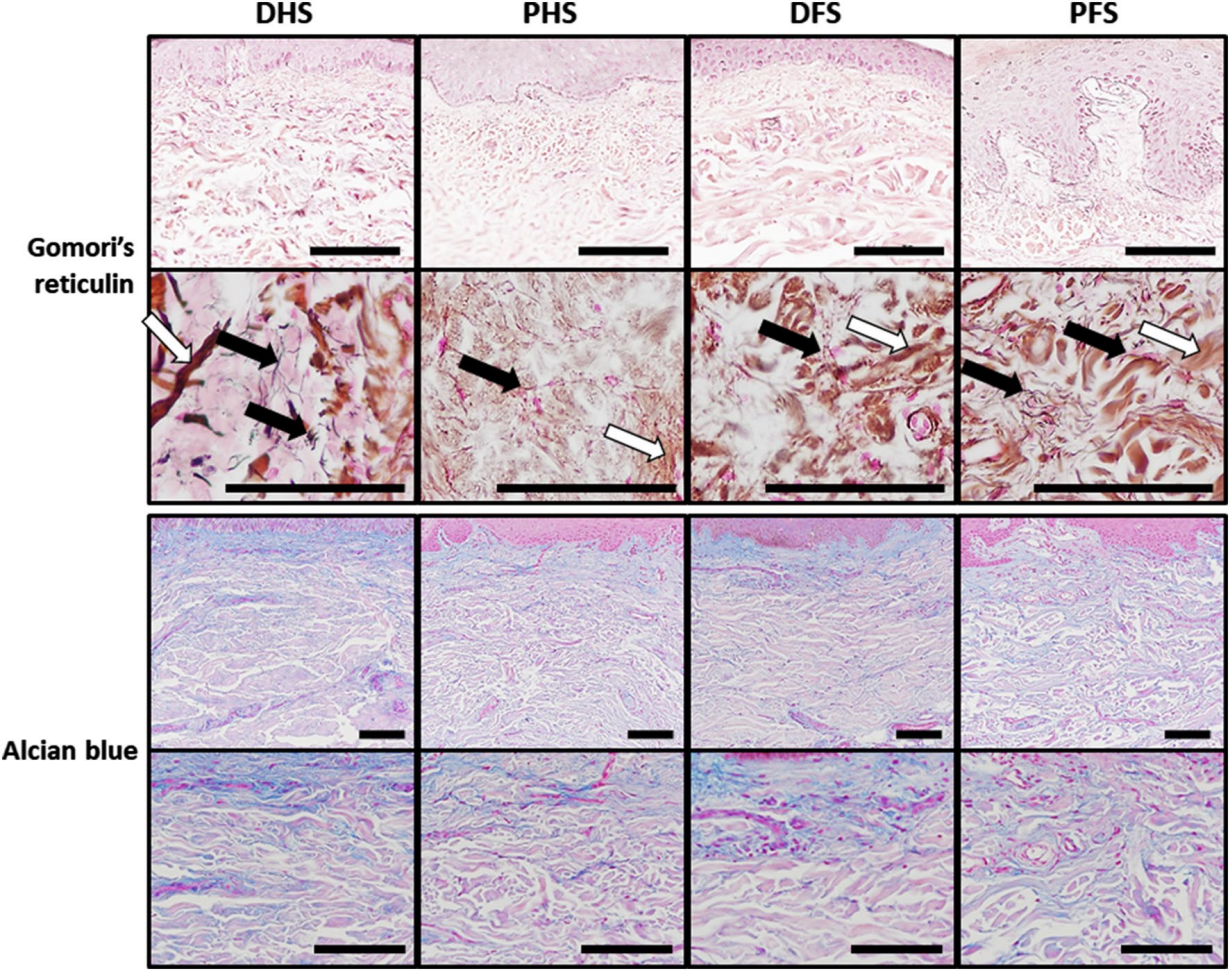
Histochemical analysis of reticular fibers and proteoglycans in the dermis of the four skin types analyzed in this work as determined by the Gomori’s reticulin and alcian blue methods, respectively. For Gomori’s reticulin, reticular fibers can be detected as thin black filaments (black arrows) that did not show significant differences among samples, although their presence was more abundant at dermal papillae suggesting that they could play an important role as basement membrane components. This method also stained some dermic collagen fibers in brown (white arrows). Similarly, very few differences were found for the alcian blue method, which stains acid proteoglycans of the dermis in blue color. *DHS* dorsal hand skin, *PHS* palmar hand skin, *DFS* dorsal foot skin, *PFS* plantar foot skin. Top panels correspond to low-magnification images of each skin type, whereas lower panels show higher magnifications of the same sample type. Scale bars: 100 µm

Quantitative histochemical analyses allowed us to quantify the main fibrillar and non-fibrillar components of the human skin dermis. First, the FMP method used to detect mature collagen fibers (Fig. 5; Table 3) showed that approximately one-third to half of the dermis area was occupied by collagen, with PFS showing significantly higher percentage of area than PHS. In addition, we found that the staining intensity was significantly higher in foot skin as compared to hand skin, and in ridged skin as compared to non-ridged skin (*p* < 0.05), with PFS having the highest intensity of the four skin types. Second, we determined the presence of elastic fibers in the dermis of each sample using orcein histochemistry (Fig. 5; Table 3). Results showed that DHS had significantly higher percentage of area occupied by elastic fibers than PHS and that DFS, but no differences were found for the elastic fibers intensity. Then, results obtained using Gomori’s reticulin metal reduction technique (Fig. 6; Table 3) showed that the staining intensity was very low in the ECM of the dermis of all samples, with no statistically significant differences among samples, although these fibers were more abundant at the rete ridges close to a papilla. Despite reticular fibers were rare in the ECM, we found these fibers at specific locations such as the basement membrane and the wall of the dermis blood vessels. Finally, the quantitative histochemical analysis of dermal acid proteoglycans (Fig. 6; Table 3) revealed that the percent of area occupied by proteoglycans ranged between 26.7 ± 21.4 and 20.2 ± 13.9 of the total dermal surface, and no statistical differences were detected among groups.

**Table 3:** Histochemical quantification of collagen, elastic and reticulin fibers and proteoglycans in the four types of skin dermis

	PA collagen fibers	Intensity collagen fibers	PA elastic fibers	Intensity elastic fibers	PA reticulin	Intensity reticulin	PA proteoglycans	Intensity proteoglycans
Dorsal hand skin (DHS)	37.0 ± 21.0	125.0 ± 4.7	14.9 ± 14.0	199.5 ± 2.0	1.9 ± 0.5	18.0 ± 12.1	26.3 ± 26.8	187.2 ± 5.3
Palmar hand skin (PHS)	35.7 ± 9.1	130.7 ± 4.1	4.9 ± 3.2	200.9 ± 2.0	1.4 ± 0.6	29.8 ± 24.1	23.9 ± 16.6	185.1 ± 8.0
Dorsal foot skin (DFS)	36.4 ± 16.8	135.8 ± 2.5	4.0 ± 2.2	197.8 ± 4.6	1.3 ± 0.6	27.7 ± 12.9	26.7 ± 21.4	185.5 ± 6.2
Plantar foot skin (PFS)	48.5 ± 11.6	140.3 ± 5.2	4.9 ± 3.6	199.8 ± 4.0	2.0 ± 0.8	30.2 ± 34.7	20.2 ± 13.9	188.3 ± 5.5
Hand skin	36.3 ± 15.6	127.9 ± 5.2	9.9 ± 11.1	200.2 ± 2.0	1.7 ± 0.6	23.9 ± 19.5	25.1 ± 20.4	186.1 ± 6.6
Foot skin	43.0 ± 15.1	138.3 ± 4.7	4.5 ± 3.0	198.9 ± 4.3	1.6 ± 0.8	29.0 ± 25.5	23.2 ± 17.5	187.0 ± 5.9
Non-ridged skin	36.7 ± 18.2	131.0 ± 6.6	8.9 ± 10.7	198.6 ± 3.7	1.6 ± 0.6	22.9 ± 13.1	26.6 ± 23.2	186.3 ± 5.7
Ridged skin	43.4 ± 12.2	136.5 ± 6.7	4.9 ± 3.4	200.3 ± 3.3	1.7 ± 0.8	30.0 ± 29.1	22,05 ± 12.1	187.0 ± 6.6
Hand skin vs. foot skin *p* value	0.2368	0.0000*	0.0759	0.5152	0.3569	0.4901	0.1557	0.5944
Ridged skin vs. non-ridged skin *p* value	0.3802	0.0339*	0.2664	0.1320	0.9568	0.7556	0.5011	0.5011
DHS vs. PHS *p* value	1.000	0.0346*	0.0346*	0.2049	0.3310	0.4051	1.000	0.3980
DFS vs. PFS *p* value	0.1135	0.0377*	0.7920	0.3066	0.4850	0.6497	0.5529	0.0750
DHS vs. DFS *p* value	0.7217	0.0004*	0.0206*	0.7217	0.0991	0.0819	0.7217	0.7217
PHS vs. PFS *p* value	0.0204*	0.0004*	1.000	0.6429	0.2233	0.7051	0.1720	0.4397

For each element, the percentage of area (PA) occupied by each component and the intensity of the signal shown by each component (intensity) are shown as average ± standard deviation. The last rows correspond to statistical *p* values for the comparison of two specific skin types

*DHS* dorsal hand skin, *PHS* palmar hand skin, *DFS* dorsal foot skin, *PFS* plantar foot skin

* * p* values below 0.05 are labeled with asterisks

#### Immunohistochemical characterization of skin dermis components

**Fig. 7:**
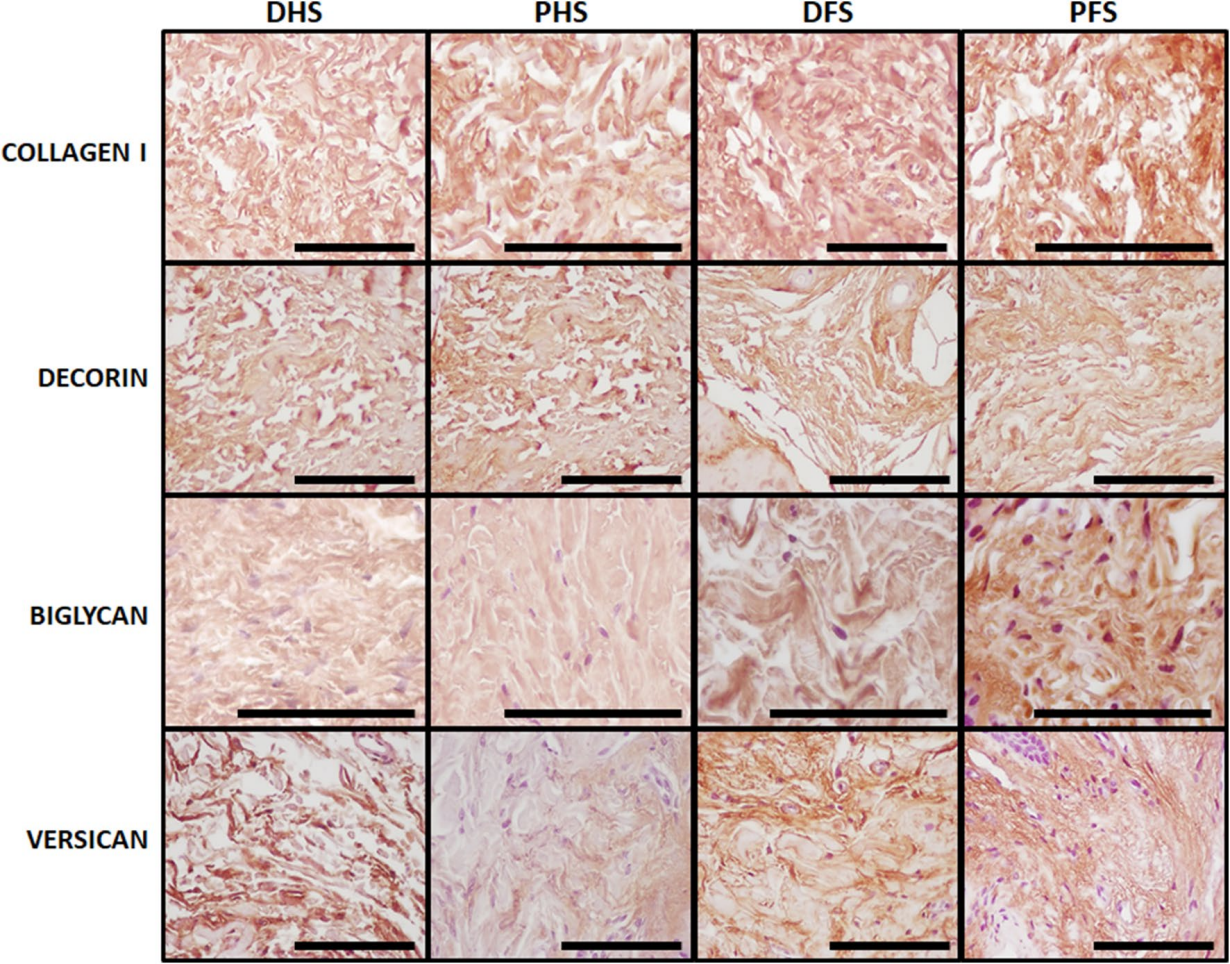
Immunohistochemical analysis of collagen I, decorin, biglycan and versican in the dermis of the four skin types analyzed in this work. All images correspond to high magnification images of the dermal layer of each type of skin. Positive reaction to each specific marker is detectable in brown. For collagen I and decorin, all samples showed mild signal, except PFS that had strong signal for collagen I. Biglycan was slightly positive in DHS and PHS, mild in DFS and strong in PFS. Versican expression was mild for all samples except for PHS, where signal was slight. *DHS* dorsal hand skin, *PHS* palmar hand skin, *DFS* dorsal foot skin, *PFS* plantar foot skin. Scale bars: 100 µm

In the first place, our immunohistochemical analysis of type-I collagen fibers (Fig. 7; Table 2) showed that these fibers were very abundant in the dermal layer of both the ridged and the non-ridged skin, with the dermis of the four types of skin analyzed here being highly positive for this protein (++), although PFS showed the highest positive signal of all samples (+++). Then, the analysis of specific proteoglycans as determined by immunohistochemistry (Fig. 7; Table 2) suggests that the small proteoglycan decorin had similar distribution and amount (++) in the dermis of the four skin types, with no differences among groups. However, biglycan was more abundant in foot skin (++/+++) than in hand skin (+) and in PFS (+++) than in DFS (++), suggesting that foot ridged skin had higher amount of this proteoglycan than the rest of skin types analyzed here. The large proteoglycan versican was expressed at similar levels by DHS, DFS and PFS (++), although showed slightly lower expression (+) in PHS samples.

#### Analysis of blood and lymphatic vessels

**Fig. 8:**
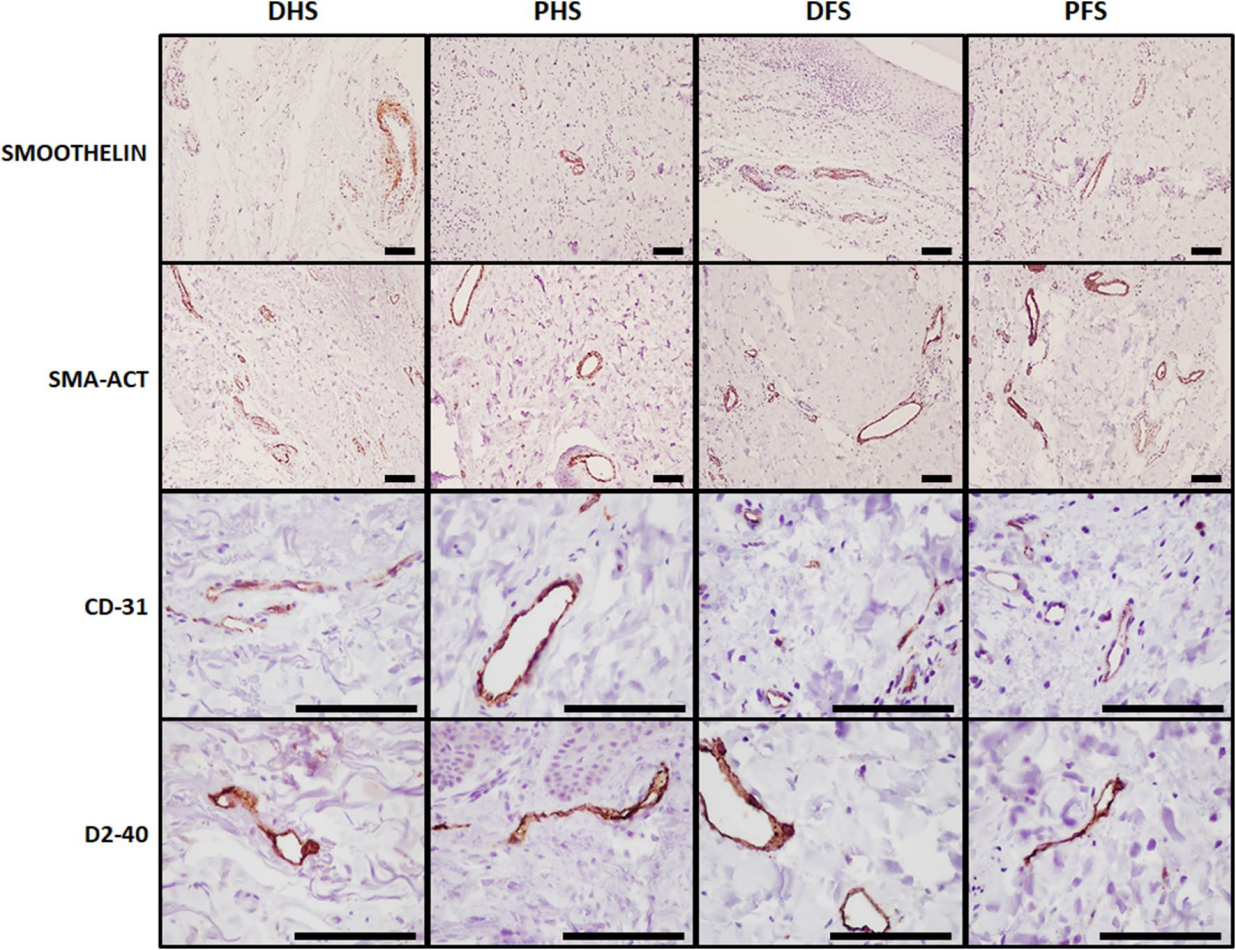
Immunohistochemical analysis of blood and lymphatic vessels in the dermis of the four skin types analyzed in this work. Blood vessels (in brown) were identified using anti-smoothelin, SMA-ACT and CD-31 antibodies and lymphatic vessels (in brown) were detected with anti-D2-40 antibodies. No significant differences were found among the four skin types analyzed in this work. *DHS* dorsal hand skin, *PHS* palmar hand skin, *DFS* dorsal foot skin, *PFS* plantar foot skin. Scale bars: 100 µm

To identify blood vessels in the dermis of each skin samples, we used immunohistochemical methods for smoothelin, SMA-ACT and CD-31 (Fig. 8). Results showed that the dermis layer of all skin types had abundant blood vessels, with no differences among skin types, and that these structures had higher diameter at the reticular dermis than at the papillary dermis. Interestingly, thin vessels showed positive signal for SMA-ACT and CD-31, whereas thick vessels allocated at the deep dermis plexus were positive for smoothelin.

Analysis of lymphatic vessels using D2-40 immunohistochemistry confirmed the presence of these vessels in the dermis of all skin types, with no differences among samples. However, vessels tended to show open internal lumen in non-ridged skin (DHS and DFS) and showed a narrow lumen in ridged skin (PHS and PFS).

### Characterization of the human skin basement membrane by immunohistochemistry and electron microscopy

**Fig. 9:**
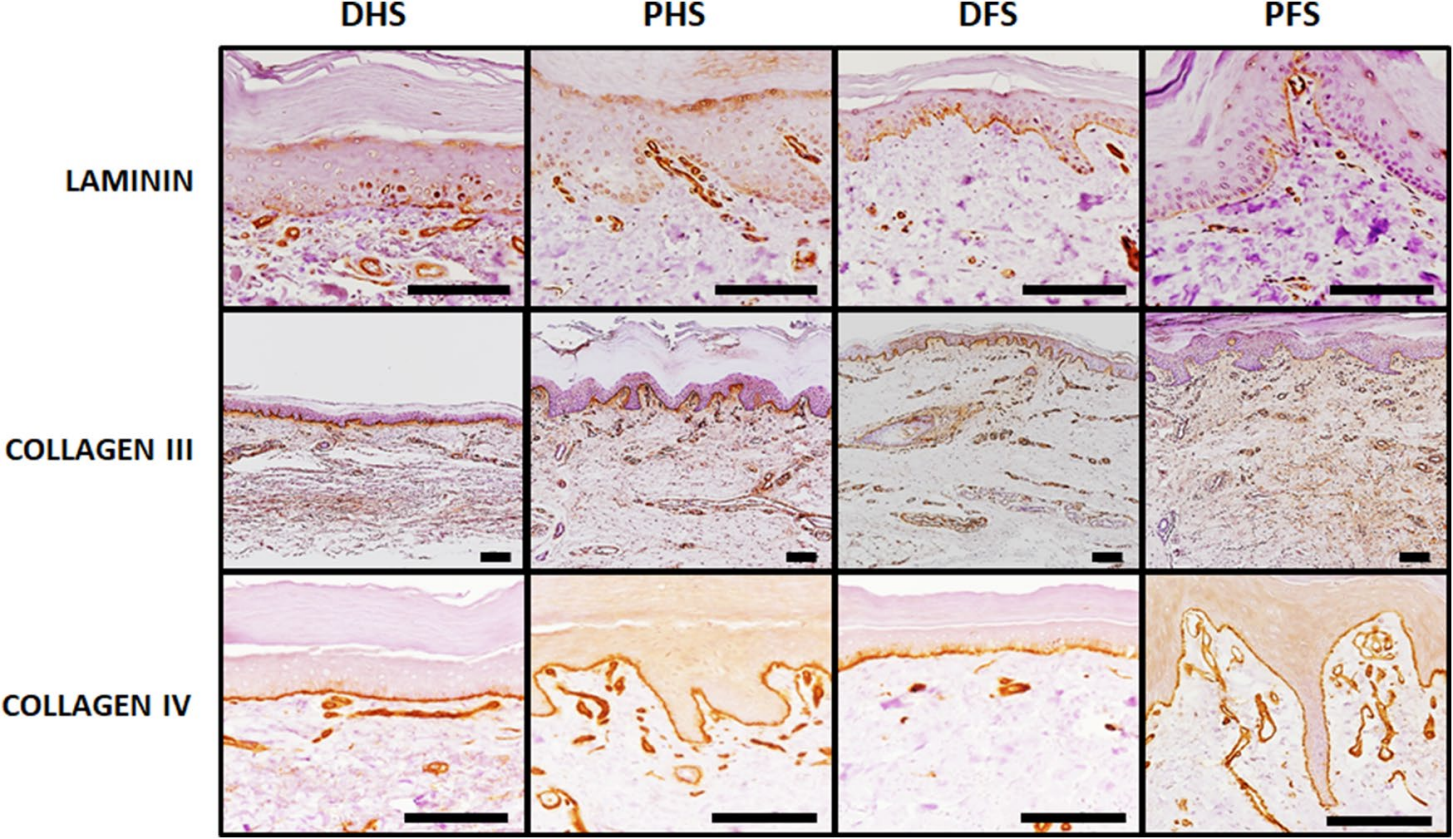
Analysis of fundamental basement membrane components at the dermo-epidermal interface of the four skin types analyzed in this work. Immunohistochemical analyses were used for the identification of laminin, collagen III and collagen IV, showing slight laminin signal (in brown) for DHS and PHS and mild signal for DFS and PFS. Collagen III and IV were strongly positive in all samples. Scale bars: 100 µm

Analysis of the main components of the skin basement membrane using immunohistochemical methods (Fig. 9; Table 2) showed positive expression of laminin, collagen III and collagen IV in the dermo-epidermal junction of all skin types, with no differences among samples. Differences among samples were minimal for collagen III and IV—strong (+++) expression for all skin types. However, laminin expression tended to be more intense (++) in foot skin (DFS and FPS) as compared to hand skin (+) (DHS and PHS).

**Fig. 10:**
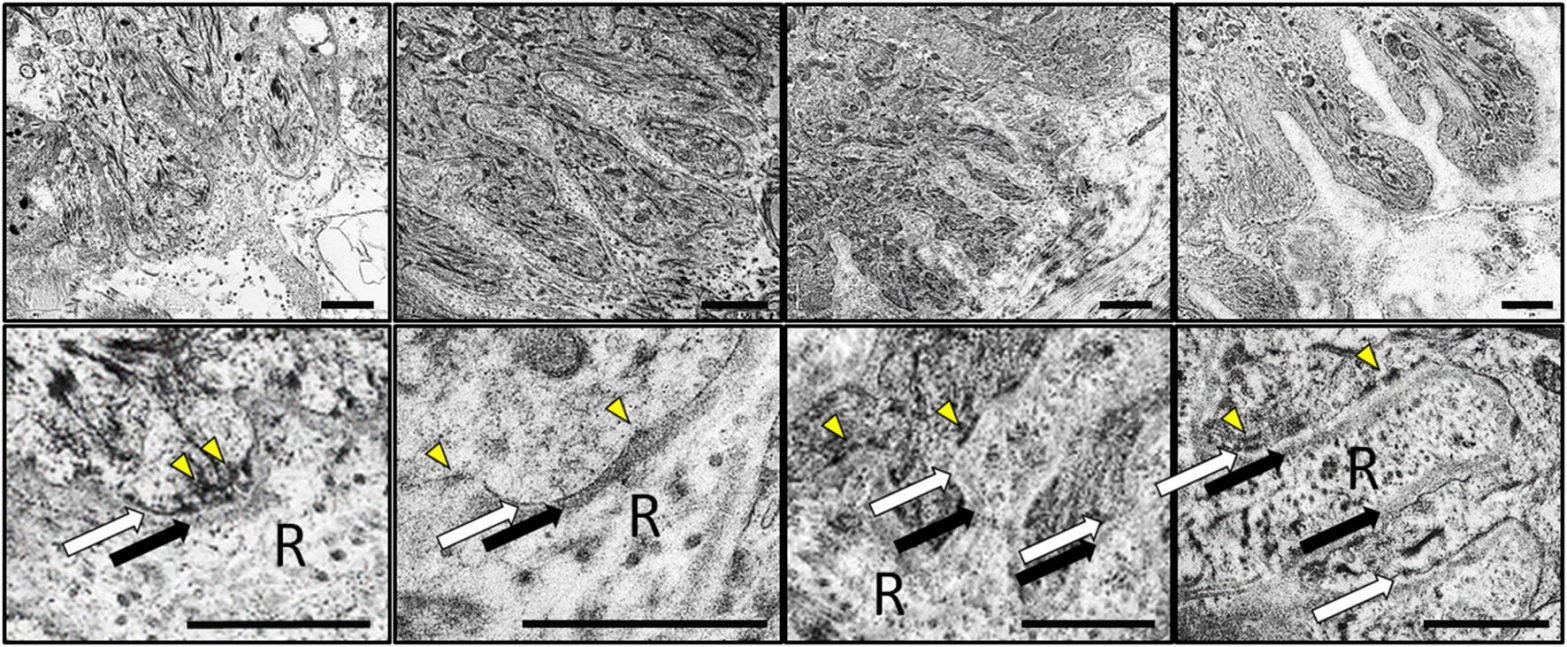
Analysis of the basement membrane of the four skin types analyzed in this work using transmission electron microscopy (TEM). Images correspond to different magnifications of the dermo-epidermal interface showing the major components of the basement membrane: the basal lamina consisting of a lamina lucida (white arrows) and a lamina densa (black arrows) and the lamina reticularis (R) containing anchoring fibrils. Illustrative hemidesmosomes have been highlighted with yellow arrowheads. *DHS* dorsal hand skin, *PHS* palmar hand skin, *DFS* dorsal foot skin, *PFS* plantar foot skin. Scale bars: 1 µm

Transmission electron microscopy analysis (Fig. 10) showed a well-structured basement membrane at the dermo-epidermal junction of all skin types, with very few differences among samples. In all cases, the basement membrane consisted of a basal lamina showing an electron-lucent lamina lucida and an electron-dense lamina densa and a lamina reticularis containing numerous anchoring fibrils. Scattered hemidesmosomes were present in all samples (Fig. 10).

## Discussion

A proper knowledge of the histological structure of the ridged skin as compared to non-ridged skin is a first step for a better understanding of the pathologic conditions affecting these organs. In the present work, we have carried out one of the first studies comparing both types of skin from the same individuals using an array of histological, histochemical and immunohistochemical methods, and we found that non-ridged and ridged skin had several differences at both the epidermis and the dermis layers. The fact that samples corresponded to the same donors, contributed to control confounding variables such as the age, gender or race that may influence the results. In general, our results revealed very few or no differences among the different skin donors analyzed in this work when the same skin type was analyzed. However, the high heterogeneity of the donors might influence our results, since it is well known that significant differences may exist between different age groups. Future works should be carried out to determine specific features of the skin corresponding to specific groups of age.

First, our analysis confirmed that ridged palmoplantar skin has a significantly thicker epidermis than non-ridged skin. These results are in agreement with previous reports stating that the depth of human ridged skin from hands and feet is 400–600 µm, whereas the non-ridged skin is in the range of 75–150 µm (Murphree 2017). Interestingly, our detailed layer-by-layer analysis showed that most layers of the epithelium were thicker in palmoplantar skin than in non-ridged skin, with the only exception of the basal layer, which consisted of a single cell layer with similar size and cell number in both skin types. Although our study only determined the bi-dimensional area of cells instead of 3D cell volume, we found that the differences among layers are mainly due to a higher size of the keratinocytes and corneocytes in ridged skin, and not only to a higher number of cells. This phenomenon has not been described before, and some authors state that the number of epithelial cell layers is the only parameter affecting the epidermis thickness (Geneser 1986). We may hypothesize that the higher pressure supported by ridged skin, which is usually thicker than non-ridged skin, could induce a process of cell pseudohypertrophy that could favor resistance of these cells. This finding is in agreement with reports demonstrating that non-ridged skin epithelium transferred to ridged skin dermis might experience a series of structural changes and would become similar to ridged skin epithelium (Yamaguchi et al. 2001). Future studies should determine cell volume to confirm these results.

On the other hand, the immunohistochemical analysis of other types of epithelial cells showed that palmoplantar skin might have less number of melanocytes, as previously suggested (Uemura et al. 2016), higher number of Merkel cells as demonstrated by (Lacour et al. 1991) and less number of Langerhans cells. This latter finding was not previously described. This coincides with a higher presence of melanin in non-ridged skin as determined by the FMP method. From a biological point of view, a possible explanation could be that the palmoplantar skin is exposed to low dosages of UV irradiation, what would make unnecessary the presence of large melanocytic populations and higher melanin production. In fact, it has been demonstrated that melanocytes tend to block their migration into ridged skin during embryonic development (Yamaguchi et al. 2004). Interestingly, different authors suggest that the influence of subjacent dermal fibroblasts is a crucial factor determining the number or the functional activity of melanocytes in ridged skin epidermis by dermo-epidermal interaction (Yamaguchi et al. 1999, 2004; Biedermann et al. 2015; Hasegawa et al. 2008). In addition, the thicker epithelial barrier found at palmoplantar skin would not require the presence of abundant immune cells such as Langerhans cells, since external antigens would not easily reach the basal and spinous epidermal strata where these cells tend to reside. Regarding Merkel cells, their function as skin mechanoreceptors makes them more necessary in ridged skin, which is subjected to continuous external forces and stimuli as compared to non-ridged skin (Munde et al. 2013). Future studies should confirm these hypotheses.

Important cytoskeleton components of epithelial cells are the cytokeratins. In this regard, we found that all skin types analyzed in this work had comparable expression of CK5/6 at the basal layer, but ridged skin had some expression at suprabasal layers. This could imply that the number of cells with proliferation potential could be higher in ridged skin, which would retain proliferation capability at suprabasal layers, and not only at the basal layer. Furthermore, all samples were negative for CK7, which is typical of simple epithelia and should not be present in normal skin epidermis (Lee and Ro 2015). Finally, CK10, a marker of terminal keratinization, was expressed at suprabasal layers of all skin types, pointing out the possibility that no keratinization differences exist between ridged and non-ridged skin. Although we did not analyze CK9 in the present work, it has been previously established that CK9 is a typical marker of ridged skin and could indeed be used to differentiate the palmoplantar skin epidermis from other types of skin in formalin-fixed samples (Knapp et al. 1986; Uemura et al. 2016; Yamaguchi et al. 1999).

The immunohistochemical analysis of intercellular junctions proteins revealed that all the samples analyzed in this work had comparable expression levels of claudin-1 and desmoplakin 1/2, although this latter tended to appear preferentially in the most apical cells of the stratum spinosum. These results point out the possibility that ridged and non-ridged skin have efficient barrier functions, with no differences between skin types, and that the formation of mature desmosomes associated to desmoplakin proteins is restricted to the most differentiated spinous cells, whereas corneodesmosomes were abundant only at the cornified stratum as previously demonstrated (Ishida-Yamamoto et al. 2017, 2018). A similar phenomenon was found for the two markers of terminal differentiation of stratified, keratinized epithelia that we analyzed here: filaggrin and involucrin. Both were more intensely expressed by the most apical epithelial cell layers, confirming the terminally differentiated status of these epithelial cell layers. The fact that ridged skin had higher number of cell layers and larger cell area may be related to our finding showing a more diffuse signal in ridged skin epidermis and that the signal was slightly more intense in non-ridged epidermis. Interestingly, some reports previously suggested that keratohyalin granules are heterogeneous and bicomponent in human skin. In fact, keratohyalin consists of a major electron-dense component containing filaggrin and a less electron-dense minor component without filaggrin (Günzel et al. 1991; Kastl and Anton-Lamprecht 1990). Although we were not able to analyze keratohyalin granules in our samples, we may hypothesize that keratohyalin may be heterogeneous in normal human skin, especially in ridged skin, as suggested. Future studies should analyze these components in deep, including comprehensive TEM studies of all cell layers of the skin.

When the dermal layer of the human skin was analyzed, we found some differences among the four skin types. One of the most important dermal component playing an important role on skin physiology and biomechanical properties is the fibrillar component, which consists of a network of different types of collagens, elastic and reticular fibers (Hussain et al. 2013; Kanitakis 2002). Collagen forms the main structural body for the skin dermis, and we found that all skin types analyzed in our work had abundant amounts of mature collagen as determined by histochemistry and collagen type-I immunostaining. However, quantification allowed us to find higher collagen content in ridged dermis than in non-ridged dermis and in foot skin than in hand skin. This could be related to the specific function carried out by each type of skin, since collagen fibers are able to transmit and dissipate energy during mechanical deformation (Shah et al. 2017), thus providing mechanical resistance to collagen-rich tissues. Therefore, it is reasonable that ridged palmoplantar skin have higher collagen content than non-ridged skin and foot skin has higher collagen than hand skin. Another important fibrillar component of the human dermis playing a role on skin elasticity is the elastic fibers. These components are characterized by long-range elastic extensibility to maintain the ability of the skin to return to its original shape (Hussain et al. 2013). In our study, we found that the skin type with higher elastin content was DHS, which is supposed to hold higher levels of biomechanical deformation than the other skin types due to its location. Finally, we found that the ECM of the human skin dermis has very low amounts of reticular fibers, although we found these fibers at the blood vessels and at the basement membrane of the human skin. Reticular fibers mainly contain collagen type III in association with other types of collagens, glycoproteins and proteoglycans, and are mostly found at basement membranes, surface of adipocytes, myocytes and Schwann cells, hepatic sinusoids and at the fibrous reticulum of lymphoid tissues (Ushiki 2002). As demonstrated by previous reports, reticular fibers are not commonly found in the ECM of the normal human skin dermis, but they may appear in pathological conditions (Antunes et al. 1999). Interestingly, we did found that reticular fibers were more abundant at dermis papillae; these fibers could correspond to reticular fibers of the lamina fibroreticularis of the basement membrane.

Proteoglycans are non-fibrillar components of the human dermis that interact with other matrix proteins such as the collagen network resulting in the formation of supramolecular structures able to increase tissue stiffness (Lee et al. 2016) and to regulate important cell functions, such as proliferation, migration, protein synthesis or degradation (Maquart and Monboisse 2014). In general, we found that all skin types had similar amounts of proteoglycans as determined by histochemistry, but our analysis of specific molecules revealed some interesting differences for biglycan, which was more abundantly expressed by foot skin. Biglycan is a small leucine-rich dermatan sulfate proteoglycan that plays an important role in the maintenance of the structural and functional integrity of connective tissues by regulating collagen and controlling tensile strength (Mizumoto et al. 2017; Godoy-Guzman et al. 2018). Therefore, the abundance of this extracellular matrix component in foot skin could contribute to its increased biomechanical properties allowing this kind of skin to withstand greater stress forces than other skin types (Swensson et al. 1998). However, these results should be taken cautiously, since some proteoglycans may be altered or lost during fixation and tissue processing.

Finally, we analyzed the basement membrane of the different skin types. The cutaneous basement membrane zone is a highly specialized functional complex that provides the skin with structural adhesion and resistance to shearing forces (Bruckner-Tuderman and Has 2014). In general, the ultrastructure -with a basal lamina and a lamina reticularis or fibroreticularis with abundant anchoring fibrils rich in reticular fibers—and the distribution of laminin and type IV—collagen of the basement membrane was very similar in all samples analyzed in the present work. The fact that no differences were found for these components of the basement membrane, and that hemidesmosomes were equally present in all skin types suggests that these important structures did not differ among the ridged and non-ridged skin found at the human hands and feet. This is in agreement with previous works published by Tidman and Eady demonstrating that the body region had no apparent influence on numbers of hemidesmosomes and other basal structures of the human skin (Tidman and Eady 1984). However, our analysis showed that laminin was more abundant in foot skin as compared to hand skin. Although these results should be confirmed by future independent studies, we may hypothesize that the intense tension forces to which the foot skin is subjected could induce laminin synthesis without altering the thickness and ultrastructure of the basement lamina at these areas.

In summary, our results suggest that the ridged and non-ridged skin found at the human hands and feet share many structural and molecular similarities. However, in the present work we demonstrated that a number of differences exist at the epithelial, dermal and basement membrane levels, and that these differences should be related to the different biomechanical forces associated to each skin type. These findings could contribute to a better knowledge of the human skin histology and could be useful for the future generation of bioartificial substitutes of each type of human skin by tissue engineering techniques.

## Electronic supplementary material

Below is the link to the electronic supplementary material.

**Supplementary Table S1**. Primary antibodies used for the immunohistochemical detection of specific components of the human dermis and conditions used for the immunohistochemical reaction. (DOCX 13 KB)
