# Supplementary material for: Characterization of the human ridged and non-ridged skin: a comprehensive histological, histochemical and immunohistochemical analysis
Source: Histochem Cell Biol. 2018 Aug 11;151(1):57–73. doi: 10.1007/s00418-018-1701-x (PMC6328512; doi:10.1007/s00418-018-1701-x)
Supplement: Supplementary file 1 — Supplementary Table S1. Primary antibodies used for the immunohistochemical detection of specific components of the human dermis and conditions used for the immunohistochemical reaction. (DOCX 13 KB) [file 418_2018_1701_MOESM1_ESM.docx]

| **Antibodies** | **Dilution** | **Pretreatment** | **Reference** |
| --- | --- | --- | --- |
| Rabbit anti-cytokeratin 5-6 | Ready-to-use | Citrate buffer, pH 6, 95ºC  for 25 min | Master Diagnostica, Granada, Spain, cat. Nº. MAD-210651Q |
| Mouse anti-cytokeratin 10 | Ready-to-use | Citrate buffer, pH 6, 95ºC  for 25 min | Master Diagnostica, Granada, Spain, cat. Nº. MAD-000150QD |
| Mouse anti-cytokeratin 7 | Ready-to-use | Citrate buffer, pH 6, 95ºC  for 25 min | Master Diagnostica, Granada, Spain, cat. Nº. MAD-001004QD |
| Rabbit anti-cytokeratin 20 | 1:100 | Citrate buffer, pH 6, 95ºC  for 25 min | Abcam, Cambridge, UK, cat Nº ab76126 |
| Mouse anti-filaggrin | 1:50 | Citrate buffer, pH 6, 95ºC  for 25 min | Abcam, Cambridge, UK, cat Nº ab17808 |
| Mouse anti-involucrin | Ready-to-use | Citrate buffer, pH 6, 95ºC  for 25 min | Master Diagnostica, Granada, Spain, cat. Nº. MAD-000164QD |
| Mouse anti-claudin-1 | Ready-to-use | Citrate buffer, pH 6, 95ºC  for 25 min | Master Diagnostica, Granada, Spain, cat. Nº. MAD-000523QD |
| Mouse anti-desmoplakin 1-2 | Ready-to-use | Citrate buffer, pH 6, 95ºC  for 25 min | Acris, Herford, Germany, cat Nº AM09122SU-N |
| Mouse anti-melan-A | Ready-to-use | EDTA, pH 8, 95ºC for 25 min | Master Diagnostica, Granada, Spain, cat. Nº. MAD-001767QD |
| Rabbit anti-CD-1A | Ready-to-use | Citrate buffer, pH 6, 95ºC  for 25 min | Master Diagnostica, Granada, Spain, cat. Nº. MAD-000673QD |
| Mouse anti-laminin | 1:1000 | Citrate buffer, pH 6, 95ºC  for 25 min | Sigma-Aldrich, Steinheim, Germany, cat. no. L8271 |
| Mouse anti-colagen IV | Ready-to-use | EDTA, pH 8, 95ºC for 25 min  Pepsin, 37ºC for 5 min | Master Diagnostica, Granada, Spain, cat. Nº. MAD-000733QD |
| Mouse anti-colagen I | 1:200 | EDTA, pH 8, 95ºC for 25 min | Acris, Herford, Germany, cat Nº R1038 |
| Rabbit anti-colagen III | 1:250 | Citrate buffer, pH 6, 95ºC  for 25 min | Abcam, Cambridge, UK, cat Nº ab7778 |
| Goat anti-decorin | 1:500 | Chondroitinase, 37ºC, 60 min | R&D System, cat Nº AF143 |
| Rabbit anti-biglycan | 1:100 | Citrate buffer, pH 6, 95ºC  for 25 min | Abcam, Cambridge, UK, cat Nº ab49701 |
| Rabbit anti-versican | 1:100 | Chondroitinase, 37ºC, 60 min | Abcam, Cambridge, UK, cat Nº ab19345 |
| Mouse anti-smothelin | Ready-to-use | EDTA, pH 8, 95ºC for 25 min | Master Diagnostica, Granada, Spain, cat. Nº. MAD-000445QD |
| Mouse anti-SMA-ACT | Ready-to-use | Citrate buffer, pH 6, 95ºC  for 25 min | Master Diagnostica, Granada, Spain, cat. Nº. MAD-001195QD |
| Mouse anti-CD-31 | Ready-to-use | EDTA, pH 8, 95ºC for 25 min | Master Diagnostica, Granada, Spain, cat. Nº. MAD-002048QD |
| Mouse anti-D2-40 | Ready-to-use | EDTA, pH 8, 95ºC for 25 min | Master Diagnostica, Granada, Spain, cat. Nº. MAD-000402QD |
